# Supplementary material for: Use of In Vitro Dynamic Colon Model (DCM) to Inform a Physiologically Based Biopharmaceutic Model (PBBM) to Predict the In Vivo Performance of a Modified-Release Formulation of Theophylline
Source: Pharmaceutics. 2023 Mar 9;15(3):882. doi: 10.3390/pharmaceutics15030882 (PMC10058579; doi:10.3390/pharmaceutics15030882)
Supplement: Supplementary file 1 [file pharmaceutics-15-00882-s001.zip › pharmaceutics-2249919-supplementary.pdf]

Use of In Vitro Dynamic Colon Model (DCM) to Inform a Physiologically Based Biopharmaceutic Model (PBBM) to Predict the In Vivo Performance of a Modified-Release Formulation of Theophylline

Supplementary material

Table S1. Theophylline PBPK model input parameters (values derived from Abduljalil et al 2022

Parameter	Value (unit)	Reference
Molecular Weight	180.2 g/mol	[1]
log P	-0.02	
Compound Type	Ampholyte	
pKa 1 (acid), pKa2 (base)	8.8, 0.99	
B/P	0.815	
Main plasma binding protein	Human serum albumin	
Fraction unbound in plasma, fu	0.5	
**Absorption**		
Absorption model option	ADAM (for adult)	
Intrinsic Solubility	1.976 mg/mL	
Permeability (Peff, man)	4.0673 x10^{-4} cm/s	
**Distribution Model**		
Distribution volume input type	Full PBPK	
Vss	0.37 L/kg	
Global tissue to plasma (Kp) Scalar	1.2	
**Elimination model**		
Renal Clearance	0.31 L/h	
Metabolism -option	Recombinant Enzyme kinetics	
CYP1A2 (N1-demethylation) Vmax<br>CYP1A2 (N1-demethylation) Km	2.47 pmol/min/mg protein<br>1080 uM	
CYP1A2 (N3-demethylation) Vmax<br>CYP1A2 (N3-demethylation) Km	6 pmol/min/mg protein<br>377 uM	
CYP2D6 (N3-demethylation) Vmax<br>CYP2D6 (N3-demethylation) Km	1.8 pmol/min/mg protein<br>6897 uM	
CYP1A2 (8-OH) Vmax<br>CYP1A2 (8-OH) Km	4.11 pmol/min/mg protein<br>394 uM	
CYP2D6 (8-OH) Vmax<br>CYP2D6 (8-OH) Km	4.68 pmol/min/mg protein<br>10709 uM	
CYP2E1 (8-OH) Vmax<br>CYP2E1 (8-OH) Km	40.78 pmol/min/mg protein<br>16855 uM	
CYP3A4 (8-OH) Vmax<br>CYP3A4 (8-OH) Km	0.4 pmol/min/mg protein<br>23393 uM	

1. Abduljalil, K., I. Gardner, and M. Jamei, *Application of a Physiologically Based Pharmacokinetic Approach to Predict Theophylline Pharmacokinetics Using Virtual Non-Pregnant, Pregnant, Fetal, Breast-Feeding, and Neonatal Populations.* Front Pediatr, 2022. **10**: p. 840710.
